# Cigarette smoking and DNA methylation

**DOI:** 10.3389/fgene.2013.00132

**Published:** 2013-07-17

**Authors:** Ken W. K. Lee, Zdenka Pausova

**Affiliations:** Physiology and Experimental Medicine, The Hospital for Sick Children, University of TorontoToronto, ON, Canada

**Keywords:** epigenetics, epigenome, DNA methylation, prenatal exposure, cigarette smoking

## Abstract

DNA methylation is the most studied epigenetic modification, capable of controlling gene expression in the contexts of normal traits or diseases. It is highly dynamic during early embryogenesis and remains relatively stable throughout life, and such patterns are intricately related to human development. DNA methylation is a quantitative trait determined by a complex interplay of genetic and environmental factors. Genetic variants at a specific locus can influence both regional and distant DNA methylation. The environment can have varying effects on DNA methylation depending on when the exposure occurs, such as during prenatal life or during adulthood. In particular, cigarette smoking in the context of both current smoking and prenatal exposure is a strong modifier of DNA methylation. Epigenome-wide association studies have uncovered candidate genes associated with cigarette smoking that have biologically relevant functions in the etiology of smoking-related diseases. As such, DNA methylation is a potential mechanistic link between current smoking and cancer, as well as prenatal cigarette-smoke exposure and the development of adult chronic diseases.

## INTRODUCTION

Methylation is a chemical modification of DNA that modulates transcription of genetic information from DNA to RNA and, in this manner, can influence the expression of a given phenotype (normal traits or diseases). The rate of methylation at a given DNA site is a quantitative trait regulated by a complex interplay of genetic and environmental factors. Here, we review the current understanding of this interplay, with a special focus on exposure to cigarette smoking – a common environmental factor acting throughout the human lifespan.

## DNA METHYLATION

### DEFINITION OF DNA METHYLATION

Methylation of DNA is the addition of a methyl group at the 5′ position of cytosines in CpG dinucleotides (CpGs; Rakyan et al., 2011). Cytosines in CpA, CpC, and CpT dinucleotides can also be methylated, but less frequently (Rakyan et al., 2011). In addition, methylated cytosines can oxidize and form 5-hydroxymethylcytosines (Ficz et al., 2011); this event is even less common (Dahl et al., 2011).

DNA methylation at a single CpG site within a single DNA strand is a *binary* trait – the site is either methylated or not. But experimental samples, such as DNA extracted from lymphocytes, contain a large number of DNA strands. Some of these strands are methylated and others not; therefore, DNA methylation at a single CpG site in an experimental sample is a *quantitative* trait, being a proportion of DNA strands that is methylated (Bibikova et al., 2011).

### GENOME DISTRIBUTION OF DNA METHYLATION

The CpG dinucleotides make up only about 1% of the human genome, which is less than one-fourth of the expected proportion if they were to be randomly distributed; the lower occurrence of CpGs is thought to be due to a high rate of spontaneous mutations of methylated CpGs to TpGs (Han et al., 2008). The CpG dinucleotides cluster within so-called CpG islands (CGIs), which are usually defined as 500-bp to 2-kb segments of DNA that exhibit at least 50% CG content and a ratio of observed CpGs to expected CpGs greater than 0.6 (Han et al., 2008; Rakyan et al., 2011). There are approximately 50,200 CGIs in the human genome; they are found most abundantly in gene promoters and repetitive DNA elements. The repetitive DNA elements are short and long interspersed repeats, or tandem repeats, such as mini- and microsatellites, that make up >50% of the genome (Lander et al., 2001). CGIs also exist in gene bodies (especially in exons) but these are less common (Medvedeva et al., 2010).

Not all CpGs are methylated. Bisulfite-DNA sequencing of human chromosomes indicates that up to 30% of CpGs are *unmethylated* (i.e., <20% of DNA molecules methylated) and over 40% of CpGs are *hypermethylated* (i.e., >80% of DNA molecules methylated); the remaining 30% of CpGs are methylated at an intermediary level (i.e., 20–80% DNA molecules methylated; Eckhardt et al., 2006). Gene-promoter CpGs are mainly unmethylated, while CpGs in gene bodies and repetitive DNA elements are mostly methylated (Suzuki and Bird, 2008; Maunakea et al., 2010). Finally, methylation levels are often correlated across multiple neighboring CpGs and this co-methylation is stronger for CpGs inside CGIs than outside CGIs (Eckhardt et al., 2006; Bell et al., 2011).

### ENZYMES OF DNA METHYLATION

Methylation of DNA is catalyzed by three families of DNA methyltransferases: DNMT1, DNMT2, and DNMT3 (Jeltsch, 2006a).

DNMT1 is responsible for life-long *maintenance* of DNA methylation during cell division – it copies DNA methylation marks from the original to the nascent strand during DNA replication (Tang et al., 2009). Consistent with this role, DNMT1 is expressed ubiquitously in proliferative cells (Jurkowska et al., 2011); it has a 30- to 40-fold binding preference for hemi-methylated than unmethylated CpGs (Jeltsch, 2006b). DNMT1 does not appear to have a specific target DNA sequence (Jeltsch, 2006a). Instead, through its interaction with the DNA replication machinery, DNMT1 is tethered to the replication fork where it methylates DNA using a hemi-methylated template (Jurkowska et al., 2011).

DNMT2 catalyzes methylation of small RNA molecules, instead of DNA, but it has some residual *de novo* DNA methyltransferase activity (Hermann et al., 2003; Jurkowski et al., 2008).

The DNMT3 family of DNA methyltransferases has three members: DNMT3A, DNMT3B, and DNMT3L. DNMT3A and DNMT3B are *bona fide de novo *methyltransferases that methylate DNA without a template (Tang et al., 2009). DNMT3L lacks DNA methyltransferase activity but it co-localizes with DNMT3A and DNMT3B and enhances their activity (Hata et al., 2002; Jurkowska et al., 2011). In contrast to DNMT1, which maintains life-long expression in proliferative cells, DNMT3A and DNMT3B are expressed mainly during early embryogenesis (though they have some specialized functions in adulthood, such as maintaining self-renewal of hematopoietic stem cells (Tadokoro et al., 2007), cooperating with DNMT1 in silencing tumor suppressor genes in colorectal cancer cells (Rhee et al., 2002), or regulating synaptic plasticity in the brain (Feng et al., 2010a; LaPlant et al., 2010). Also in contrast to DNMT1, DNMT3A and DNMT3B appear to have preferences for certain DNA sequences. For example, they prefer CpGs flanked by upstream purine bases and downstream pyrimidine bases (Handa and Jeltsch, 2005). In addition, they form tetrameric complexes with DNMT3L (Jia et al., 2007) and the structural orientation of active sites and fixed spacing between adjacent complexes allow simultaneous methylation of CpGs separated by 8–10 bp (Jurkowska et al., 2008, 2011). These intrinsic properties of *de novo *DNMTs may influence how DNA methylation patterns are established during early embryogenesis.

### FUNCTION OF DNA METHYLATION

The main functions of DNA methylation are the regulation of gene expression and protection of genome integrity.

#### Regulation of gene expression

Methylation of DNA can modulate transcription of DNA to RNA by influencing DNA binding of proteins that initiate and perform DNA transcription (Portela and Esteller, 2010; Shukla et al., 2011). Methylation of DNA may do so either directly, through physical impediment of protein binding, or indirectly, through chromatin remodeling and its effect on DNA accessibility for proteins that regulate DNA transcription (Portela and Esteller, 2010). DNA methylation impacts not only the quantity but also the form of produced RNA; the latter effect is mediated by DNA methylation modulating the use of alternative promoters (Maunakea et al., 2010) and splice sites (Shukla et al., 2011).

Methylation of DNA has traditionally been considered an inhibitor of DNA transcription. This effect is best understood in the context of *gene promoters* where DNA methylation can impair physical binding of transcription enhancers and promote the recruitment of methyl-CpG-binding domain proteins that increase chromatin condensation and thus decrease the DNA accessibility for the transcriptional machinery (Portela and Esteller, 2010). Consistent with this inhibitory effect of DNA methylation on DNA transcription, CpGs within 1 kb of transcription start sites are generally hypomethylated (Bell et al., 2011; Jjingo et al., 2012). The inverse correlation of DNA methylation and gene expression may also depend on CGI context; CpG sites outside of CGIs are two times more likely to be correlated with gene expression than CpG sites within CGIs (Numata et al., 2012).

In contrast to DNA methylation in *gene promoters*, DNA methylation in *gene bodies* has been associated with enhanced DNA transcription (Aran et al., 2011; McGowan et al., 2011). Gene-body DNA methylation is common in ubiquitously expressed genes and correlates positively with gene expression at a genome-wide level (Hellman and Chess, 2007; Suzuki and Bird, 2008; Ball et al., 2009). It has been proposed that, rather than a cause of enhanced gene expression, gene-body DNA methylation may be its consequence (Zilberman et al., 2007; Jjingo et al., 2012). During early embryogenesis – when DNA methylation patterns are being established – an open chromatin structure of chromosomal regions containing actively transcribed genes allows DNMTs to access and methylate DNA. Within these regions, CpGs in promoters of actively transcribed genes are occupied by DNA-binding transcription factors and cannot be methylated, but CpGs in gene bodies that are not occupied by these factors can and are methylated (Jjingo et al., 2012). This pattern of DNA methylation (low in promoters and high in gene bodies) established during early embryogenesis is then copied throughout life and, through low promoter methylation, may contribute to higher expression of these genes (Jeltsch, 2006a; Ball et al., 2009). Assuming this happens for a large number of genes, gene-body methylation then correlates positively with DNA transcription at a genome-wide level (Jjingo et al., 2012). In addition to this indirect positive relationship between gene-body methylation and genome-wide gene expression, gene-body methylation may be involved in suppression of DNA transcription directly by, for example, inhibiting alternative promoters embedded in gene bodies (Maunakea et al., 2010), or by impeding RNA-polymerase transit and transcription elongation (Zilberman et al., 2007; Deaton et al., 2011).

#### Protection of genome integrity

Methylation of DNA protects the genome’s integrity by suppressing mobility of transposable elements (TEs). TEs are repetitive DNA sequences that have the ability to integrate into new chromosome locations, through either “cut-and-paste” or “copy-and-paste” mechanisms (Levin and Moran, 2011). TEs, like non-transposable repetitive elements, are hypermethylated, resulting in their transcriptional silencing (Law and Jacobsen, 2010). Since the transposition machinery requires TE-encoded enzymes, suppression of TE transcription by DNA methylation effectively prevents translocations and gene disruptions (Levin and Moran, 2011). Loss of DNA methylation allows for TE reactivation and transposition (Tsukahara et al., 2009).

### DNA METHYLATION CHANGES DURING DEVELOPMENT AND AGING

**FIGURE 1:**
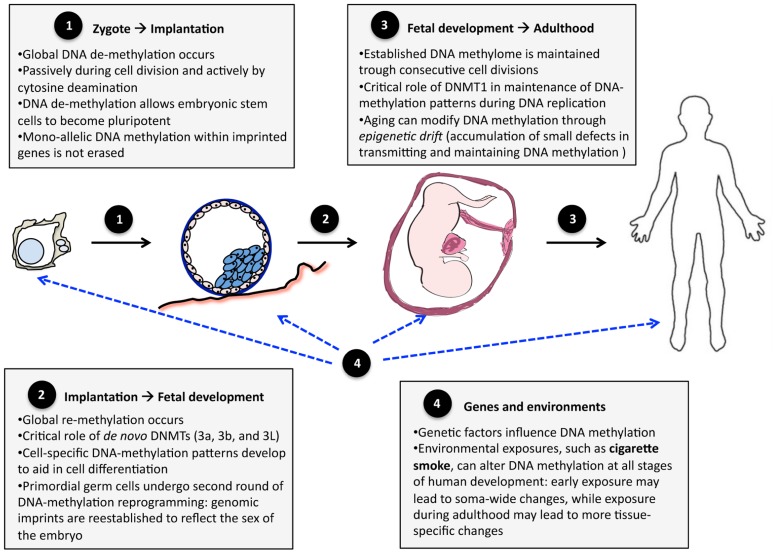
**DNA methylation during development and aging.** The changes in the human methylome can be divided into three stages: (1) transition from zygote to pre-implantation blastocyst, (2) transition from implanted blastocyst to early fetal development, and (3) transition into adult life. Genes and the environment (4) influence DNA methylation patterns at each of these stages.

During embryogenesis, DNA methylation is a highly dynamic trait (Eckhardt et al., 2006; Talens et al., 2010; **Figure 1**). Immediately after fertilization, the zygote genome undergoes global depletion of DNA methylation, reaching its lowest levels at the pre-implantation-blastocyst stage – the stage when pluripotent embryonic stem cells form most of the inner cell mass of the embryo (Feng et al., 2010b). Post-implantation, DNA methylation patterns are re-established and become relatively stable and similar to those found in adult somatic cells (Smith et al., 2012). Mono-allelic DNA methylation within imprinted genes escapes the global erasure/re-establishment to preserve parent-of-origin expression (Bartolomei, 2009). Furthermore, the cells that differentiate into primordial germ cells undergo an additional round of global erasure/re-establishment to reflect the sex of the embryo (Feng et al., 2010b). In both somatic and primordial germ cells, the global erasure of DNA methylation is mediated by cytosine deaminases (Bhutani et al., 2010; Popp et al., 2010) and the re-establishment of DNA methylation patterns is catalyzed mainly by *de novo* DNA methyltransferases, DNMT3A and DNMT3B (Okano et al., 1999; Kato et al., 2007).

The erasure/re-establishment of DNA methylation during early embryogenesis aids in the process of cell differentiation (Epsztejn-Litman et al., 2008). Global erasure of DNA methylation activates the expression of pluripotency genes, which promote the development of embryonic stem cells, which are cells capable of generating any tissue in the body (Straussman et al., 2009). These pluripotency genes are re-methylated upon the initial stages of cell differentiation (Epsztejn-Litman et al., 2008), at which point cell-specific DNA methylation patterns begin to develop (Straussman et al., 2009), enabling cells to have specific structures and functions.

After embryonic re-establishment, DNA methylation patterns are maintained by DNMT1 during successive cell divisions (i.e., DNA methylation marks are copied from original to nascent strands during DNA replication) and, as such, are relatively stable throughout life (particularly during young and middle-aged adulthood). This maintenance of DNA methylation is not perfect, however. It has been observed that older monozygotic twin pairs demonstrate greater DNA methylation differences than younger monozygotic twin pairs (Fraga et al., 2005). This so-called *epigenetic* drift might be due to the accumulation of small errors in copying DNA methylation marks during successive cell divisions (Martin, 2005). The differences between twins were greater for pairs that spent less of their lifetime together or exhibited more different lifestyles, suggesting that environmental factors play a role in *epigenetic drift* (Fraga et al., 2005). In addition, senescence is associated with global demethylation of the genome (Bjornsson et al., 2008; Bollati et al., 2009), possibly due to decreasing DNMT1 activity (Lopatina et al., 2002).

### TISSUE-SPECIFICITY OF DNA METHYLATION PATTERNS

A considerable similarity in DNA methylation levels exists across different tissues. By bisulfite-DNA sequencing of human chromosomes in 12 different tissues, it was estimated that only about 5–15% of CpGs are methylated in a tissue-specific manner (Eckhardt et al., 2006). Furthermore, these differential CpGs are more likely to be found between developmentally distant tissues, such as liver (derived from endoderm) and kidney (derived from mesoderm; Pai et al., 2011) than between developmentally close tissues, such as various types of lymphocytes (all derived from mesoderm; Eckhardt et al., 2006).

### SEX DIFFERENCES IN DNA METHYLATION

A high degree of similarity in DNA methylation patterns also exists between males and females (Eckhardt et al., 2006; Talens et al., 2010). Nonetheless, some sex differences do exist. The most notable is the hypermethylation of gene promoters on X chromosome that occurs only in females (Hellman and Chess, 2007). This process is initiated during the blastocyst stage (coinciding with global re-methylation of autosomes); it is mediated by DNMT1 and DNMT3B (Hansen et al., 2000; Csankovszki et al., 2001), and executed at random with respect to the parental origin of the inactivated X chromosome (Escamilla-Del-Arenal et al., 2011). Another sex difference pertains to the frequency of DNA methylation aberrations, which are more frequent in male than female aborted or stillborn fetuses (Pliushch et al., 2010). DNA methylation aberrations are also more easily induced in males than females by early environments, such as prenatal exposure to cigarette smoke or a lack of folate (Hoyo et al., 2011; Murphy et al., 2012a).

Taken together, DNA methylation at any given CpG is the result of multiple processes that are orchestrated as a developmental cascade involving, first, global demethylation after fertilization, then, cell/tissue-specific re-methylation during very early embryogenesis and, finally, maintenance of DNA methylation throughout the remainder of life. Each of these processes depends on the supply of methyl groups and the catalytic activity of DNA methylation enzymes (and associated machinery), both of which are regulated by genetic and environmental factors.

## GENETIC AND ENVIRONMENTAL INFLUENCES ON DNA METHYLATION

Methylation of DNA at a given CpG is a quantitative trait regulated by a complex interplay of genetic and environmental factors. Twin and family-based studies suggest that a significant proportion of inter-individual variability in DNA methylation is determined genetically (Bjornsson et al., 2008; Kaminsky et al., 2009). DNA methylation is more similar in monozygotic than dizygotic twins (Kaminsky et al., 2009) and age-related changes in DNA methylation during adulthood show familial clustering with estimated heritability of >70% (Bjornsson et al., 2008).

### GENETIC INFLUENCES ON DNA METHYLATION

The contribution of genetic factors to DNA methylation may vary across individual CpGs. Some CpGs are located directly on single nucleotide polymorphisms (SNPs) and, if their Cs or Gs are mutated into other nucleotides, they are not methylated. In these CpGs, DNA methylation behaves as a monogenic trait, being “high” in non-mutant homozygotes, “intermediate” in heterozygotes and “low” in mutant homozygotes. Interestingly, such effects of sequence variation can spread across neighboring CpGs and thus contribute to the observed correlated nature of DNA methylation at neighboring CpGs (Bell et al., 2011). For example, at a CpG located on rs10846023 (a T/C SNP), the level of DNA methylation is highly allele-specific; methylation beta on the T allele was close to 0%, whereas methylation beta on the C allele was >60% (Shoemaker et al., 2010). This effect of allele specificity was exhibited at nearby CpGs, spanning over 500 bp (Shoemaker et al., 2010).

Further support for DNA methylation being in part determined genetically comes from genome-wide association studies (GWAS) testing genotype–phenotype associations between >600,000 SNPs and DNA methylation at >25,000 CpGs (Bell et al., 2011; Numata et al., 2012). These GWAS identified a large number of SNPs (~3,000) associated with the level of DNA methylation at various CpGs; most of them were located within 2-kb regions of interrogated CpGs, but some were further apart or even on different chromosomes (Bell et al., 2011; Numata et al., 2012). Of note, given the large number of statistical tests typically performed in a GWAS of DNA methylation (i.e., 600,000 SNPs × 25,000 CpGs) and the need for correction for multiple comparisons, only SNPs with very large effects (explaining >20% of variance at a given CpG) have been reported, thus leaving out undiscovered SNPs with smaller but likely biologically meaningful effects.

Other than creating or abolishing a CpG by mutation, the mechanisms of how DNA variants modulate DNA methylation are not well understood. The results of the above GWAS suggest that some mechanisms may be regional, whereas others may be more global, or even genome-wide. The SNPs associated with DNA methylation at nearby CpGs are likely to exert regional effects; these may be related to specific sequence variants interfering with the action of the DNA methylation machinery (Handa and Jeltsch, 2005). For example, *in vitro* studies suggest that the *de novo* methyltransferases DNMT3A and DNMT3B may have intrinsic preferences for certain flanking sequences (Jurkowska et al., 2011) and that the DNMT3A/DNMT3L complex favors methylation of CpGs distributed periodically and separated by distances of 8–10 bp (Jia et al., 2007). The SNPs associated with DNA methylation at multiple distant CpGs located across large regions and on different chromosomes are likely exerting more global effects; these may be related to sequence variants modulating the expression or catalytic activity of enzymes involved in the process of DNA methylation. For example, a recent GWAS identified a SNP associated with global level of DNA methylation, which was located in the gene encoding disco-interacting protein 2 homolog B (*DIP2B*; Bell et al., 2011); this protein contains a DNA methyltransferase 1-associated protein 1-binding domain and, as such, may be part of the DNA methylation machinery (Winnepenninckx et al., 2007). In the same study, a weaker association with global DNA methylation levels was found near *DNMT1*, which encodes the key enzyme for DNA methylation maintenance (described in Section “DNA Methylation”; Bell et al., 2011). Apart from GWAS, candidate-gene studies revealed additional genes associated with global DNA methylation level; these were the methylenetetrahydrofolate reductase gene (*MTHFR*; Castro et al., 2004), which encodes an enzyme involved in the generation of methyl groups required for DNA methylation (Foley et al., 2009), and *DNMT3B* (Murphy et al., 2012b), which is one of the two key *de novo* DNA methyltransferases (described in Section “DNA Methylation”).

### ENVIRONMENTAL INFLUENCES ON DNA METHYLATION

Methylation of DNA may also be modified by environmental factors (Terry et al., 2011). Environments acting during early embryogenesis (e.g., when global erasure and re-establishment of DNA methylation occur) may induce extensive, *soma-wide *modifications of DNA methylation, whereas environments acting later during life are more likely to induce less extensive, *tissue-specific* modifications of DNA methylation (**Figure 1**). The former may be involved in fetal programing of adult disorders (Suter and Aagaard, 2012), whereas the latter may play a role in tissue-specific carcinogenesis (for example, Ehrlich and Lacey, 2013). One environment implicated in both these effects is exposure to *cigarette smoke.*

### EXPOSURE TO CIGARETTE SMOKE

Cigarette smoking is still common, despite well-publicized adverse consequences on health (reviewed in Sherman, 1991). In Canada and the USA, for example, ~20% of all adults and ~10% of pregnant women smoke at present (Centers for Disease Control and Prevention, 2011; Health Canada, 2011; Haghighi et al., 2013). It is well established that (1) *active cigarette smoking* is a major risk factor of cancer, cardiovascular disease, and chronic obstructive pulmonary disease (Kannel et al., 1987; Bartecchi et al., 1994; Doll et al., 1994) and (2) *prenatal exposure to cigarette smoke* causes fetal growth restriction prenatally, increases risk for sudden infant death syndrome postnatally and promotes the development of addictive behavior, immune-system abnormalities, obesity and associated cardiometabolic diseases postnatally (Power et al., 2010; Syme et al., 2010; Winans et al., 2011; Haghighi et al., 2013). Part of these effects may be mediated through cigarette smoke-induced modulations of DNA methylation.

**FIGURE 2:**
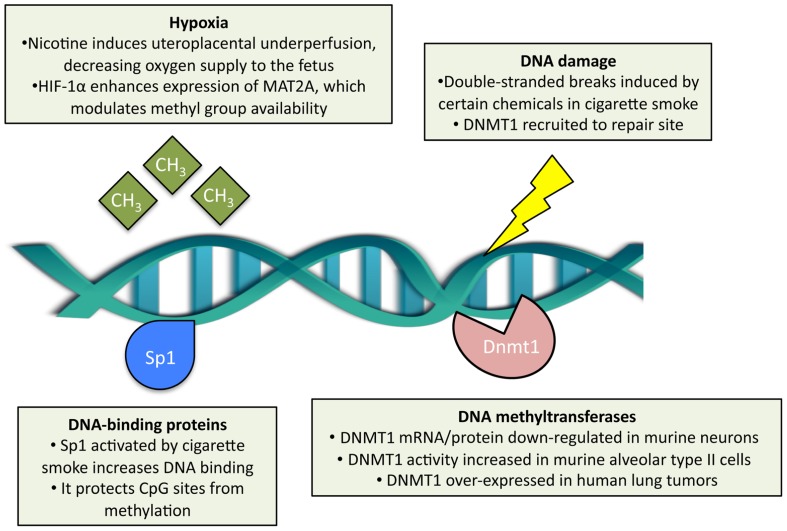
**Effects of cigarette smoke exposure on DNA methylation.** Cigarette smoke has been shown to modulate DNA methyltransferase 1 (DNMT1) content, both at the transcript and protein level, and enzymatic activity separately in different cell types. Double-stranded DNA breaks may be induced by cigarette smoke, which subsequently recruits DNMT1 adjacent to the repair site. DNA-binding proteins, such as Sp1, are activated by cigarette smoke and protect CpG sites from *de novo* methylation. In the context of prenatal exposure, cigarette smoke induces hypoxia in the embryo, which in turn modulates methyl group availability.

Cigarette smoke is considered one of the most powerful environmental modifiers of DNA methylation (Breitling et al., 2011). The specific mechanisms of how cigarette smoke may alter DNA methylation are becoming better understood (**Figure 2**). First, cigarette smoke may modulate it through *DNA damage* and subsequent recruitment of DNMTs. Carcinogens in cigarette smoke, such as arsenic, chromium, formaldehyde, polycyclic aromatic hydrocarbons, and nitrosamines (Smith and Hansch, 2000; Suter et al., 2010), can damage DNA by causing double-stranded breaks, as shown in mouse embryonic stem cells exposed to cigarette-smoke condensate (Huang et al., 2012). In these experiments, survivor cells display a high capacity for DNA repair and normal karyotypes (Huang et al., 2012). The DNA repair sites recruit DNMT1 (Mortusewicz et al., 2005), which methylates CpGs adjacent to the repaired nucleotides (Cuozzo et al., 2007). Second, cigarette smoke may also modulate DNA methylation through *nicotine* effects on gene expression (Lee and D’Alonzo, 1993). Nicotine binds to and activates the nicotinic acetylcholine receptors (present abundantly in the central and peripheral nervous systems) and thus increases intracellular calcium and leads to downstream activation of cAMP response element-binding protein, a key transcription factor for many genes (Shen and Yakel, 2009). Acting possibly through this pathway, nicotine has been shown to downregulate DNMT1 mRNA and protein expression in mouse brain neurons (Satta et al., 2008). Third, cigarette smoke may alter DNA methylation indirectly through the modulation of expression and activity of *DNA-binding factors*. It has been demonstrated, for example, that cigarette-smoke condensate increases Sp1 expression and binding to DNA in lung epithelial cells (Mercer et al., 2009; Di et al., 2012). Sp1 is a common transcription factor that binds to GC-rich motifs in gene promoters (Kadonaga et al., 1987) and plays a key role in early development; as such, it may prevent *de novo* methylation of CpGs within these motifs during early embryogenesis (Han et al., 2001). Fourth, cigarette smoke may alter DNA methylation via *hypoxia* – cigarette smoke contains carbon monoxide that binds to hemoglobin (competitively with oxygen) and thus decreases tissue oxygenation (Olson, 1984). Hypoxia, in turn, leads to the HIF-1α-dependent upregulation of methionine adenosyltransferase 2A, which is an enzyme that synthesizes *S*-adenosylmethionine, a major biological methyl donor critical for DNA methylation processes (Liu et al., 2011).

Below we review studies investigating the alterations of DNA methylation associated with current and prenatal exposures to cigarette smoke. We focus mainly on studies employing epigenome-wide technologies (see **Box 1**).

**Box 1. Methods of measuring DNA methylation.**

**Locus-specific DNA methylation**

• Bisulfite pyrosequencing (Frommer et al., 1992): Detects DNA methylation at single-CpG resolution over a short span of DNA (<1 kb) and requires only a small amount of DNA for analysis. It is a quantitative sequencing method used for a pre-selected genomic region.

**Locus-independent global DNA methylation**

• Bisulfite pyrosequencing of repetitive DNA elements (Breton et al., 2009): Methylation of repetitive DNA sequences, such as long interspersed elements (LINEs), short interspersed elements (SINEs), and satellite DNA, have been used as markers of global DNA methylation, as these elements are abundantly found throughout the genome.

• ELISA-based methylation assay (Guerrero-Preston et al., 2010): Uses methylcytosine-specific antibodies to quantify the relative DNA methylation between samples.

• [^3^H]-methyl acceptance assay (Terry et al., 2008): Relies on *SssI* prokaryotic methylase to incorporate [^3^H]-labeled methyl groups at all unmethylated CpGs. Amount of incorporated [^3^H] is inversely proportional to endogenous DNA methylation.

• Luminometric methylation assay (LUMA; Karimi et al., 2006): Relies on genomic cleavage by methylation-sensitive and -insensitive restriction enzymes; difference in amount of cleavage between these two types of enzymes is a readout for global methylation.

**Epigenome-wide DNA methylation**

• Reduced representation bisulfite sequencing (RRBS; Gu et al., 2011): Uses next-generation sequencing technology on bisulfite-converted DNA fragments, enriched at CpG-dense regions of the genome to reduce amount of sequencing required.

• Illumina 450K Methylation BeadChip (Dedeurwaerder et al., 2011): A robust high-throughput methylation microarray, providing coverage to over 480,000 CpG sites across 96% of CpG islands and 99% of RefSeq genes across the genome.

• Illumina GoldenGate Assay for Methylation (McRonald et al., 2009): This platform utilizes pre-selected CpG sites within genes relevant for specific diseases or pathways, such as the GoldenGate Methylation Cancer Panel I, which spans 1,505 CpG loci from 807 cancer-related genes.

### CURRENT EXPOSURE TO CIGARETTE SMOKE AND DNA METHYLATION

Several epigenome-wide studies have examined whether cigarette smoking is associated with modifications of DNA methylation. One of the first ones was conducted with the Illumina 27K Methylation BeadChip, which interrogates DNA methylation at >27,000 CpG sites located mostly in gene promoters (Breitling et al., 2011). This study, conducted with DNA from peripheral lymphocytes, identified a differentially (smokers vs. non-smokers) methylated CpG site in the protease-activated receptor 4 gene (*F2RL3*), achieving Bonferroni-corrected significance threshold (*p* < 1.81 × 10^-^^6^) for multiple testing of >27,000 CpGs (Breitling et al., 2011). At this site, DNA methylation was significantly lower in smokers than non-smokers (% methylation difference = 12%; *p* = 2.7 × 10^-^^31^) and correlated negatively with the number of smoked cigarettes and positively with the duration of smoking abstinence (Breitling et al., 2011). Similar exposure-related differences in the methylation of this gene were also seen in another independent study (% methylation difference = 8%; *p* = 8.4 × 10^-^^11^; Shenker et al., 2012). Interestingly, *F2RL3* is involved in platelet activation and intimal hyperplasia and inflammation – DNA methylation changes in this gene may represent a mechanistic link between cigarette smoking and cardiovascular disease (Breitling et al., 2011).

More recent studies have been conducted with the Illumina 450K Methylation BeadChip, which interrogates >450,000 CpG sites located not only in gene promoters but also in gene bodies and intergenic regions (Sandoval et al., 2011). These studies replicated the previous *F2RL3* findings and identified further CpG sites methylated differentially between smokers and non-smokers. Among the latter ones, most significant were those found in the body of the aryl hydrocarbon receptor repressor gene (*AHRR*; Monick et al., 2012; Shenker et al., 2012). At these sites, DNA methylation was significantly lower in smokers than non-smokers in two different tissues, at a Bonferroni-corrected significance threshold of *p* < 10^-^^7^, namely the lungs (% methylation difference = 34%; *p* = 1.97 × 10^-^^9^) and peripheral lymphocytes (% methylation difference = 17%; *p* = 2.3 × 10^-^^15^; Monick et al., 2012; Shenker et al., 2012). When tested in the lungs, DNA methylation correlated inversely with the levels of *AHRR* mRNA in both smokers and non-smokers (Monick et al., 2012). *AHRR* encodes a transcription factor that inhibits the aryl hydrocarbon receptor pathway, which enhances the expression of detoxification (xenobiotic-metabolizing) enzymes of environmental pollutants, such as polycyclic aromatic hydrocarbons contained in cigarette smoke (Opitz et al., 2011). Thus, cigarette smoking-induced decreases in *AHRR* DNA methylation and related increases in *AHRR* expression may compromise the body’s capacity to metabolize and thus remove harmful environmental chemicals, and as such may represent a potential mechanism of increased risk of carcinogenesis in smokers.

Finally, it should be noted that cigarette smoking may alter DNA methylation in multiple tissues (Hammons et al., 1999; Peters et al., 2007; Suzuki et al., 2007; Satta et al., 2008), and some of these alterations may differ between the tissues. Most of the current investigations, however, utilized DNA from peripheral lymphocytes or buccal cells only (due to the ease of their sampling) and, thus, did not examine this potential tissue variability. Furthermore, several epigenome-wide studies of disease outcomes closely related to cigarette smoking have been conducted (e.g., lung cancer; Carvalho et al., 2012 and chronic obstructive pulmonary disease; Qiu et al., 2012), but they did not examine effects of cigarette smoking *per se*. As such, these studies are outside the scope of the present review. Epigenetic signatures of disease outcomes closely related to cigarette smoking have been reviewed recently in Sundar et al. (2011).

### PRENATAL CHRONIC EXPOSURE TO CIGARETTE SMOKE AND DNA METHYLATION

Cigarette smoke may influence the fetus in a number of ways, some of which may also alter DNA methylation. The latter include certain chemicals in cigarette smoke (e.g., carcinogenic xenobiotics and nicotine) that can pass through the placenta to the developing embryo and fetus (Lambers and Clark, 1996). The passage of some of these chemicals is diminished by the detoxifying capacity of placenta (i.e., it metabolizes harmful xenobiotics; Sanyal et al., 1994; Suter and Aagaard, 2012). One molecule involved in this function is cytochrome P450 (CYP1A1), which is a phase-I enzyme in a two-phase detoxifying pathway (Nebert and Dalton, 2006). Prenatal exposure to cigarette smoke has been associated with lower DNA methylation of *CYP1A1* at CpG sites surrounding the xenobiotic response-element, which is a major transcriptional enhancer of *CYP1A1* expression (Suter et al., 2010). It was also associated with higher *CYP1A1* mRNA expression (Suter et al., 2010). Gene-specific effects of prenatal exposure to cigarette smoke on placental DNA methylation and mRNA have also been seen in another study employing, side-by-side, the Illumina 27K Methylation BeadChip and the IlluminaHG-12 gene-expression array (Suter et al., 2011). The authors searched for genes in which promoter DNA methylation is correlated with mRNA expression. They identified a significantly larger number of such genes in exposed (*n* = 438) than non-exposed placentas (*n* = 25; Suter et al., 2011). Interestingly, many of these genes encoded molecules involved in hypoxia response- and oxidative stress-regulating pathways (e.g., HIF-1α signaling; Suter et al., 2011). Whether these DNA methylation (and mRNA) modifications develop as functional adaptations to the greater need to detoxify xenobiotics and/or respond to hypoxia in the exposed placentas remains to be determined.

Prenatal exposure to cigarette smoke has been associated with altered DNA methylation not only in placentas but also in offspring tissues. Studies examining DNA methylation globally showed that exposed vs. non-exposed individuals exhibit lower level of DNA methylation at birth (cord serum, ELISA-based method; Guerrero-Preston et al., 2010) as well as during childhood (buccal cells, bisulfite conversion, and pyrosequencing of DNA repetitive elements; Breton et al., 2009) and middle-aged adulthood (peripheral blood cells, [^3^H]-methyl acceptance assay; Terry et al., 2008). The most recent study of individual CpGs conducted with the Illumina 450K Methylation BeadChip suggests that exposure is associated not only with global changes but also with changes at specific CpG sites (Joubert et al., 2012). Examining DNA methylation in cells from cord blood and assessing prenatal cigarette-smoke exposure by circulating maternal cotinine (a metabolite of nicotine and a stable biomarker of cigarette smoking), the authors identified 26 epigenome-wide significant CpGs that were differentially methylated between exposed and non-exposed individuals (Joubert et al., 2012). Among these, the most significant were those located in the xenobiotic-detoxifying genes, namely *AHRR* and *CYP1A1*; as discussed above, these two genes have been shown previously to be differentially methylated by cigarette smoke. Similar to the current chronic exposure to cigarette smoke in adult smokers, the CpGs in *AHRR* showed lower DNA methylation in exposed vs. non-exposed, but in contrast to prenatal chronic exposure to cigarette smoke in placentas, the CpGs in *CYP1A1* demonstrated higher DNA methylation in exposed vs. non-exposed (Joubert et al., 2012). The differentially methylated CpGs in *CYP1A1* in cord blood were the same as those in placenta (Suter et al., 2010). The reasons for these seemingly opposite effects of prenatal exposure to cigarette smoke on DNA methylation in placental and fetal tissues are not clear at present. It is of note, however, that placental (vs. fetal) tissues are globally hypomethylated, suggesting potential differences in the regulation of DNA methylation between the two types of tissues (Santos et al., 2002; Fuke et al., 2004; Novakovic et al., 2010; Macaulay et al., 2011).

## SUMMARY AND FUTURE DIRECTIONS

Cigarette smoking continues to be a major health problem, and understanding the mechanisms of its effects is an important area of research. DNA methylation represents an epigenetic modification that can mediate the effects of cigarette smoke on gene expression, and ultimately disease-relevant phenotypes. *Prenatal chronic exposure to cigarette smoke* is an adverse environmental stimulus at a time where the DNA methylome of the offspring is highly dynamic and the genome-wide methylation patterns develop; as such, these changes are likely soma-wide and maintained throughout life. *Current cigarette smoking* occurs at a time when DNA methylation patterns are already established, but need to be properly maintained during cell divisions. Since cigarette smoke is known to modulate expression and activity of the maintenance methyltransferase DNMT1, actively dividing cells may be more susceptible to exposure-mediated defects in DNA methylation; this may in part explain the link between smoking and cancer.

Recent technological developments allow for the interrogation of CpG methylation status across the genome, and this has uncovered associated CpG sites within candidate genes including those that have not been previously implicated in cigarette smoking (Joubert et al., 2012). Remarkably, in both prenatal and current cigarette smoke exposure, similar genes, such as those involved in chemical detoxification (*AHRR*, *CYP1A1*), are differentially methylated, suggesting that smoking effects might be targeted to specific regions of the epigenome. Cigarette smoking may also have shared global consequences, as both prenatal and current exposures are associated with epigenome hypomethylation (Guerrero-Preston et al., 2010; Shigaki et al., 2012). In medicine, there is a growing practice of using the individualized genome to understand a patient’s health and disease, but interpreting such information is dependent on epigenetics, such as DNA methylation. “Epigenotyping” patients to complement genomic data may be necessary in the near future.

## Conflict of Interest Statement

The authors declare that the research was conducted in the absence of any commercial or financial relationships that could be construed as a potential conflict of interest.
